# Premature deaths by visceral leishmaniasis in Brazil investigated through a cohort study: A challenging opportunity?

**DOI:** 10.1371/journal.pntd.0007841

**Published:** 2019-12-19

**Authors:** Ana Nilce S. Maia-Elkhoury, Gustavo Adolfo Sierra Romero, Samantha Y. O. B. Valadas, Marcia L. Sousa-Gomes, José Angelo Lauletta Lindoso, Elisa Cupolillo, Jose Antonio Ruiz-Postigo, Daniel Argaw, Manuel J. Sanchez-Vazquez

**Affiliations:** 1 Communicable Diseases and Environmental Determinants of Health (CDE), Neglected, Tropical and Vector Borne Diseases (VT), Pan American Health Organization/ World Health Organization (PAHO/WHO), Duque de Caxias, Rio de Janeiro, Brazil; 2 Center for Tropical Medicine, Faculty of Medicine, University of Brasilia (UNB) Brasília, Distrito Federal, Brazil; 3 Secretary of Health Surveillance (SVS), Ministry of Health, Brasilia, Distrito Federal, Brazil; 4 Institute of Infectology Emilio Ribas and Institute of Tropical Medicine from University of São Paulo, São Paulo, Brazil; 5 Oswaldo Cruz Institute (IOC) Oswaldo Cruz Foundation (Fiocruz), Rio de Janeiro, Brazil; 6 Department of Neglected Tropical Diseases (HTM/NTD/IDM), World Health Organization (WHO), Geneva, Switzerland; 7 Pan-American Foot-and-Mouth Disease Center–PANAFTOSA/PAHO, Duque de Caxias, Rio de Janeiro, Brazil; Centro de Pesquisa Gonçalo Moniz-FIOCRUZ/BA, BRAZIL

## Abstract

**Background:**

Visceral Leishmaniasis (VL) is the most severe form of leishmaniasis because it can lead to death. In the Americas, 96% of cases are in Brazil, and despite efforts, the fatality rate has increased in the past years. We analyzed deaths associated to VL in Brazil and investigated the factors that could influence on the timeliness of fatal outcome with emphasis on time (tStoD).

**Methodology:**

The registered deaths by VL were sourced from the Brazilian National Notification System from 2007–2014. Through a retrospective cohort study, univariate and multivariable Cox proportional hazards model analysis were performed and investigated the factors that could influence the time (tStoD). These factors were analyzed through survival models.

**Results:**

Out of the 1,589 reported deaths, the median for onset of the symptoms and the case notification date (tStoN) is 25 days (10–61), and for date of case notification and death (tNotD) is 9 days (4–17). The time (tStoN) to event investigation for HIV non-infected individuals was 1.4 (1.16–1.68) greater than the HIV positive group. At the same time peri-urban and urban area were 0.83 (0.47–1.44) and 1.33 (1.16–1.52), respectively. The explorations revealed apparent differences between the time to event investigation (both for tStoN and tNotD) and the age at the onset of the symptoms. According to the tStoN the rate of notification is 1.73 times greater in patients under 5 years old at the onset of the clinical symptoms compared to older patients.

**Conclusion:**

VL patients under 5 years old were diagnosed earlier and had shorter survival. It could mean that in younger population, although properly diagnosed, the fatality pattern might be related to the severity of the disease. Main host characteristics were evaluated, and age and co-infections seem to have an impact in the disease progression.

## Author summary

Visceral Leishmaniasis (VL) is present worldwide, affecting mainly economically vulnerable populations in over 80 countries. In the Americas, Brazil accounts for approximately 96% of the cases. VL as one of the most relevant endemic neglected diseases in the world, due to its incidence and high fatality, which is generally reported among untreated individuals, and its emerging pattern in human immunodeficiency virus (HIV) infected individuals. Despite the increasing knowledge on prognostic factors, incorporation of new diagnostic tools and increasing access to new treatments, case fatality rate remains high in Brazil. The analysis revealed differences in the time to event investigation according to HIV status, being greater for HIV infected individuals, particularly for the period between case notification and death. The time between onset of the symptoms and case notification appears to be greater in peri-urban and rural areas compared to the urban areas. Also, differences in the time to event investigation according to patient’s age at the onset of symptoms was evident, being longer for 5 years old or older patients. Our findings identified that children under 5 years old are at higher risk of premature death being relevant for planning interventions aimed at reduction of VL lethality in Brazil.

## Introduction

Visceral Leishmaniasis (VL) is a vector borne disease with worldwide distribution, affecting mainly economically vulnerable populations in over 80 countries from Asia, Europe, Middle East, Africa and the Americas. The World Health Organization (WHO) estimates 200,000 to 400,000 VL cases per year around the world. However, only about 58,000 cases are notified every year, from which over 90% are concentrated in seven countries: India, South Sudan, Sudan, Ethiopia, Brazil, Somalia and Kenya [1–2]. In the Latin America, this disease has been described across 12 countries with the highest occurrence in Brazil, corresponding to approximately 96% of the cases in this region [3].

VL usually has a systemic and chronic manifestation, characterized by prolonged irregular fever, hepatosplenomegaly, lymphadenopathy, pancytopenia, hypergammaglobulinemia, hypoalbuminemia, weight loss, edema and progressive weakness that could induce to cachexia, and if left untreated it may cause death in most cases [4–6]. Thus, the WHO considers VL as one of the most relevant endemic disease in the world, due to its incidence and high fatality rate, which is generally reported primarily among untreated individuals, and to its emerging pattern in human immunodeficiency virus **(**HIV) infected individuals [7]. Diagnosis of VL is performed by the combination of epidemiological data and clinical findings in conjunction with parasitological or serological tests, especially rapid diagnostic tests (RDTs) [8–10]. Unfortunately, there are some scenarios where laboratory testing is not timely available, and the diagnosis and treatment are based on just epidemiological and clinical findings [11].

One of the main objectives of the Brazilian Surveillance and Control Program for Visceral Leishmaniasis (PVC-LV, acronym in Portuguese) is the reduction of the fatality rate through early diagnosis and treatment. For this purpose, actions toward surveillance and patient assistance were implemented, such as the use of liposomal amphotericin B as a first-choice drug for vulnerable specific groups and inclusion of RDTs, allowing a more timely, practical and accurate diagnosis [12–13].

Traditionally, factors associated to VL fatality have been the co-existence of other infectious diseases and bleeding [14–17]. However, those are late clinical signs, which do not allow the proper intervention to prevent death. Thus, it is fundamental to identify signs, symptoms or laboratorial findings of prognostic value to effectively reduce the progression to death [14–19]. In Brazil, Costa et al. developed a prognosis system based on scores allowing the early identification, through clinical and laboratory data of patients with the highest probability of dying from VL [20]. Currently, this score system is in use in the country as a criterion to decide which health care level is best suited for patient management [21].

Although VL has been known for some time, the therapeutic arsenal is limited. In Brazil, diagnosis and treatment of leishmaniasis is universally available, and meglumine antimoniate and liposomal amphotericin B are more commonly used [22–23]. Notwithstanding, the fatality rate is still high despite the efforts to assure early diagnosis and access to treatment. According to data from the WHO, in 2014, the fatality rate fluctuated from 0 to 7% worldwide [24]. In Brazil, there was a reduction of the fatality rate between 2004 and 2007; however, this percentage has grown in the last years, from 5.4% in 2007 to 7.8% in 2015 [25].

Different *Leishmania infantum* populations are detected in Brazilian endemic regions. Although these populations seem to be correlated with eco-epidemiological aspects observed in different endemic regions [26–27], so far, no association between parasite’s population or genotype and clinical aspects have been observed. During the 90’s, approximately 90% of the VL reported cases were concentrated in the Northeast Region and the disease was autochthonous in 19 States belonging to four of the five Brazilian regions [28–29]. Currently, the Northeast Region holds 59% of the confirmed VL cases, and the disease is autochthonous in 21 States and the Federal District across the five regions of the country. VL affects mainly children under 10 years old and males [12, 30]. Despite its initial classification as a rural disease, deforestation, migratory movements and population growth in the peripheral urban slums of medium and large-size municipalities have been appointed as the main determinants of the expansion and alteration of the epidemiological profile pattern in Brazil. [30].

Host’ genetic characteristics have been associated to the disease, but not to its severity. It is not clear why some individuals infected with *L*. *infantum* will remain asymptomatic while others will develop the classical VL form. It has been argued that characteristics of the parasite influences the outcome of the disease. In the Brazil, *L*. *infantum* is the recognized etiological agent of VL; nonetheless, there are also unusual clinical presentations reported in some immunocompetent individuals with dissemination of other *Leishmania* species, typically associated to cutaneous leishmaniasis, to internal organs [31].

Presently, despite the increasing knowledge on prognostic factors, effective incorporation of new diagnostic tools, and increasing access to new treatments like liposomal amphotericin B, the fatality rate, has demonstrated an increasing trend over the last years. Seeing that the 2030 Agenda for sustainable development, an outcome of the United Nations Millennium Summit held in 2015, has as one of its goals the ending of Acquired immunodeficiency syndrome (AIDS), tuberculosis, malaria and neglected tropical diseases epidemics, it is highly urgent to understand the VL fatality rate in Brazil to stop such unacceptable death [32]. The objective of this study is to analyze deaths associated to VL in Brazil, and to explore the factors that could have an influence on the timeliness of fatal outcome with emphasis on time elapsed between the onset of symptoms to death.

## Materials and methods

This is study fits a retrospective cohort approach utilizing existing information about VL surveillance in Brazil.

### Data source

All registered deaths by VL (defined as: deaths directly associated to the infection by *Leishmania infantum* or to complications of this infection) were sourced from the Brazilian National Notification System (Sinan-NET, acronym in Portuguese) from 2007 to 2014. The system holds individual information on VL cases, including deaths, such as: date of birth, onset of the first clinical signs, date of the disease notification and date of death. Additionally, we also gathered information on gender, HIV status, area of residence and ethnic group, which were also available at the Sinan-NET. A total of 1,757 deaths by VL were registered in the system for the study period; however, 168 deaths were excluded from this analysis due to data inconsistency and unavailability. Therefore, a total of 1,589 deaths by VL were analyzed in this study.

### Time to event periods and variables of interest

The time elapsed between the onset of the first symptoms of VL to death was defined, at first, as the time of the event of interest according to the objective of the study. The onset of the first symptoms of VL was defined by the presence of fever, emaciation, splenomegaly and hepatomegaly. To facilitate the investigations in a more detailed manner, we decided to split the period elapsed between the first symptoms to death into: i) period between the onset of the symptoms and the case notification date (registration date in the Sinan-NET) (tStoN); ii) period between the case notification date and death (tNotD).

The variable of interest as potential determinants influencing the time to event period were gender, HIV status, area of residence, ethnic group and year of the notification. Additionally, age at the onset of the first VL symptoms was computed calculating the time-gap between the date of birth and date of the onset of the first VL symptoms.

### Exploratory and statistical analyses

Firstly, the variables were explored using tables and graphs. Additionally, Kaplan−Meier survival estimates graphs were used to graphically explore the factors affecting the two time to event periods (tStoN and tNotD) investigated. Subsequently, the two time to event periods were analyzed in survival models to investigate the factors (i.e. gender, HIV status, area of residence, age at the onset of the first VL symptoms and year of the notification) influencing the time to outcome (notification and death). Thus, two separate multivariable Cox proportional hazard models, one for tStoN and one for tNotD, were computed to investigate the effect of the different predictive variables on the time to incident (notification or death). There were not censoring events considered in these models, since both outcomes investigated in the models (i.e. notification and death) occurred for all the observations. Given the small number of investigated predictors, all of them were attempted into a multivariable Cox proportional hazards model using a Wald stepwise process, and covariates were kept if they met a P-value <0.05 as significant criteria. Once final multivariable models were developed, validity of assumption that the effects of predictors (covariates) are constant over time for Cox proportional hazards models were tested [33]. To do so, the scaled Schönfeld [34] residuals were plotted against time together with a smooth curve that helped with the interpretation of the results.

Furthermore, as a sort of a sensitivity analysis to evaluate the consistency of the results in relation to the influence of the observation with the zero values in the two time to event periods investigated (tStoN and tNotD), the analyses were repeated with the datasets without including those observations. That is, for tStoN those in which the date of the notification was the same as the data of the onset of the symptoms, and for tNotD those in which the date of the death was the same as data of the notification. The results of these investigations are presented in the S1 Supporting Information.

All the above-mentioned analyses were performed with the R version 2.5.1 from the R Foundation for Statistical Computing. http://www.r_project.org, library (survival)

## Results

### Exploratory analysis

The 1,589 VL deaths represented a 198.6 annual mean of deaths per year (standard deviation 19.5). The number of female cases was 549 (35%) while 1,040 were males (65%). One-hundred thirty-six (8.6%) cases were HIV positive, 985 (62%) were HIV negative and for 468 cases the HIV status was not recorded. Regarding the ethnicity, nine (0.6%) cases were Asian, 151 (9.5%) black, 15 (0.9%) indigenous, 962 (61%) *mulatos*, 284 (17.9) whites and for 168 (10.6%) cases the ethnicity group was not recorded. Table 1 presents the number of the VL cases categorized according to age at the onset of the symptoms and at death, demonstrating to be very stable between these two events.

**Table 1:** Distribution of the VL number of cases included in the study according to their age at the onset of the symptoms and at death (n = 1,589).

Age groups	No. Cases at the onset of the symptoms	No. Cases at death
Under 5 years old	406	402
From 5 to 10 years old	33	37
From 10 to 20 years old	75	74
From 20 to 50 years old	530	524
Over 50 years old	527	534
Unknown	18	18

**Fig 1:**
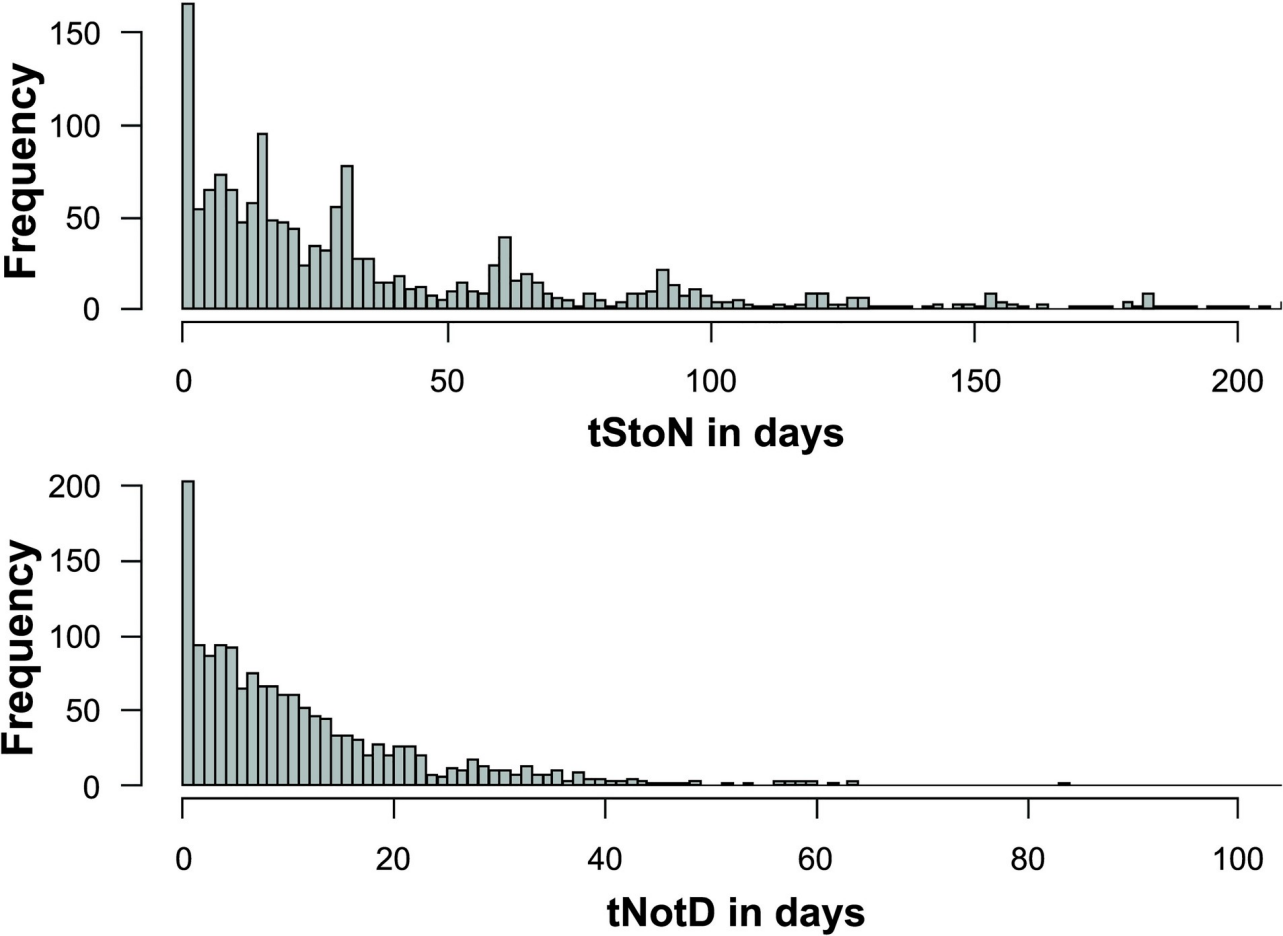
Histogram presenting the frequency distribution of the time periods from the onset of the symptoms to the notification of the disease (tStoN) (top) and from the notification to the death (tNotD) (bottom).

The histogram in Fig 1 shows the distribution of VL deaths by tStoN and tNotD. The median for tStoN is 25 days (with the interquartile range—IQR- being 10 to 61), 5^th^ percentile is 0. The median for tNotD is nine days (with the interquartile range -IQR- being 4 to 17), 5^th^ percentile is 0. In both cases it is observed a right-skewed distribution, with a peak in the zeros (also shown by the 5^th^ percentile), indicating that in many cases, the notification date is the same as the first observed symptoms of VL. Likewise, the date of the notification coincides with the date of the death.

**Fig 2:**
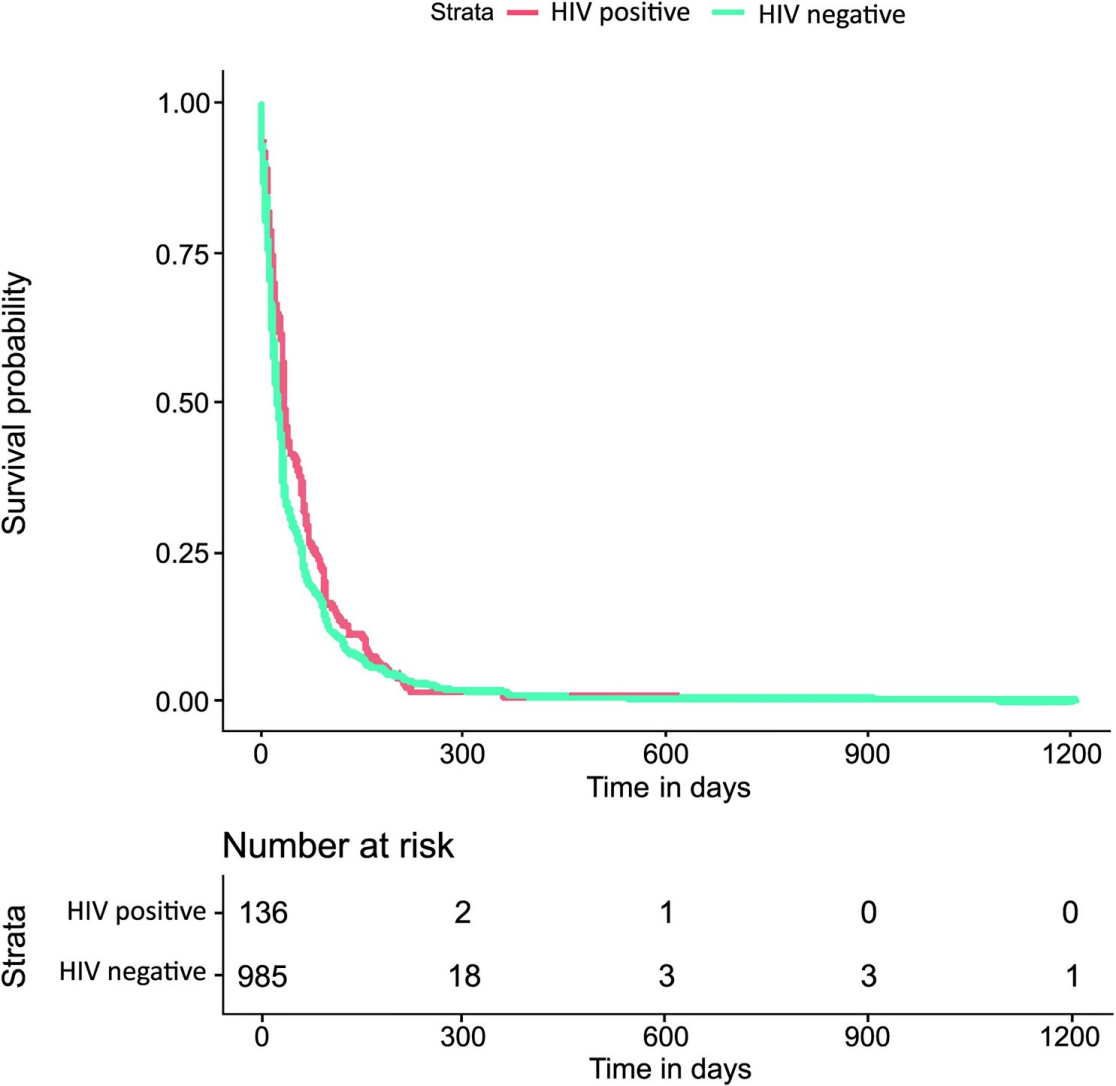
Kaplan−Meier survival estimate for the time period from the onset of the symptoms to the notification of the disease (tStoN) according to the HIV status.

**Fig 3:**
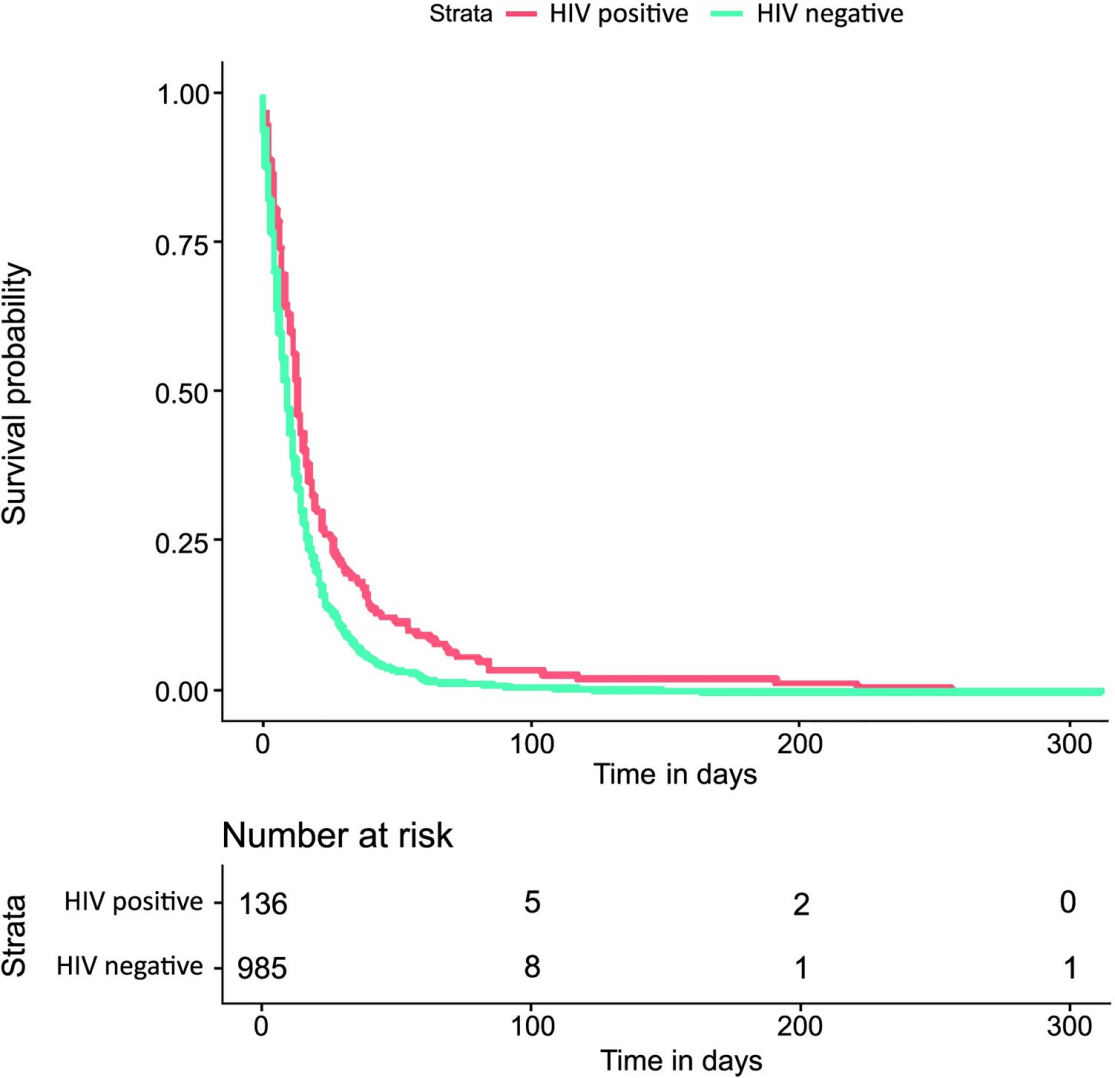
Kaplan−Meier survival estimate for the time period from the notification to the death (tNotD) according to the HIV status.

**Fig 4:**
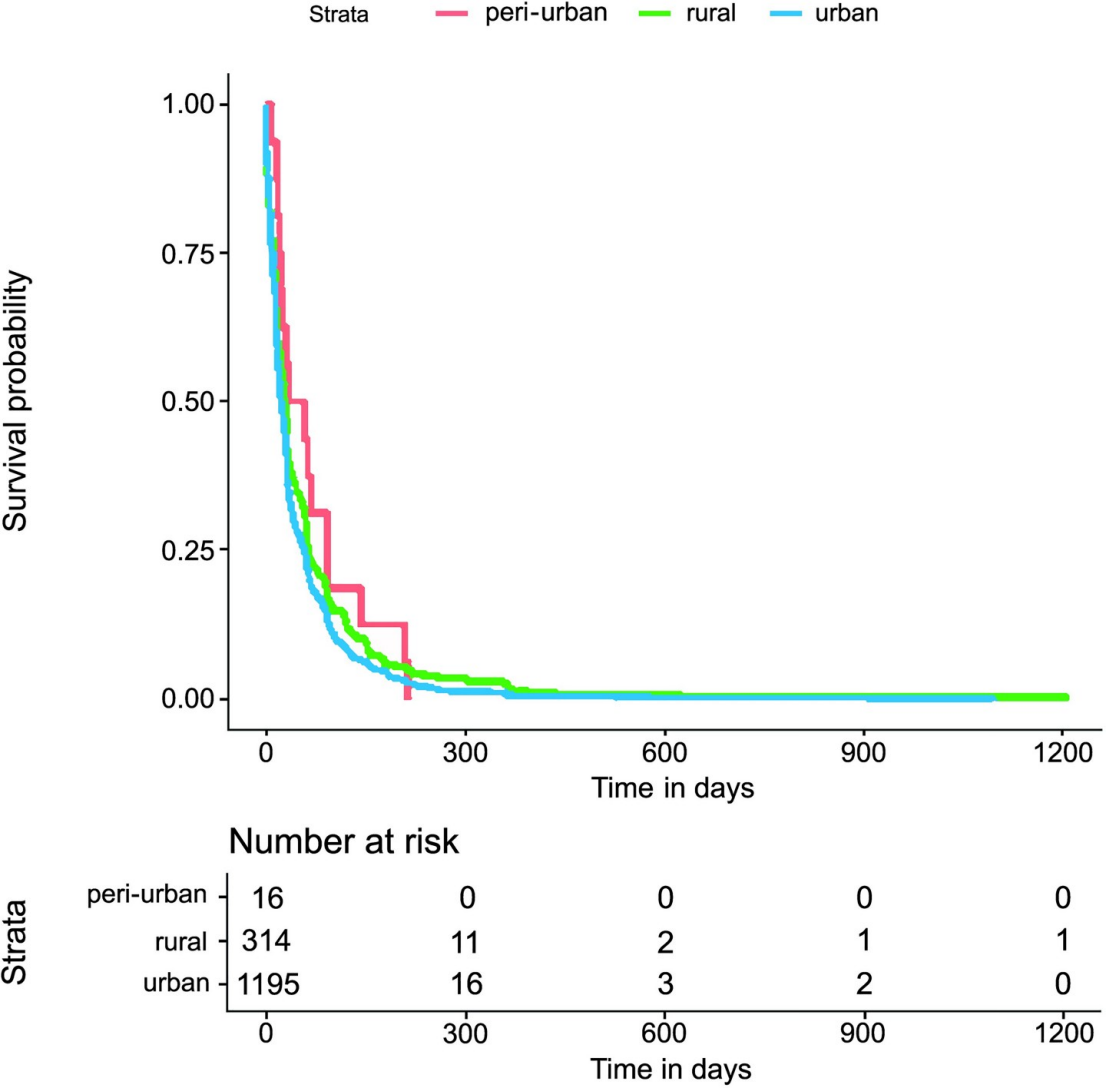
Kaplan−Meier survival estimate for the time period from the onset of the symptoms to the notification of the disease (tStoN) according to the area of living.

**Fig 5:**
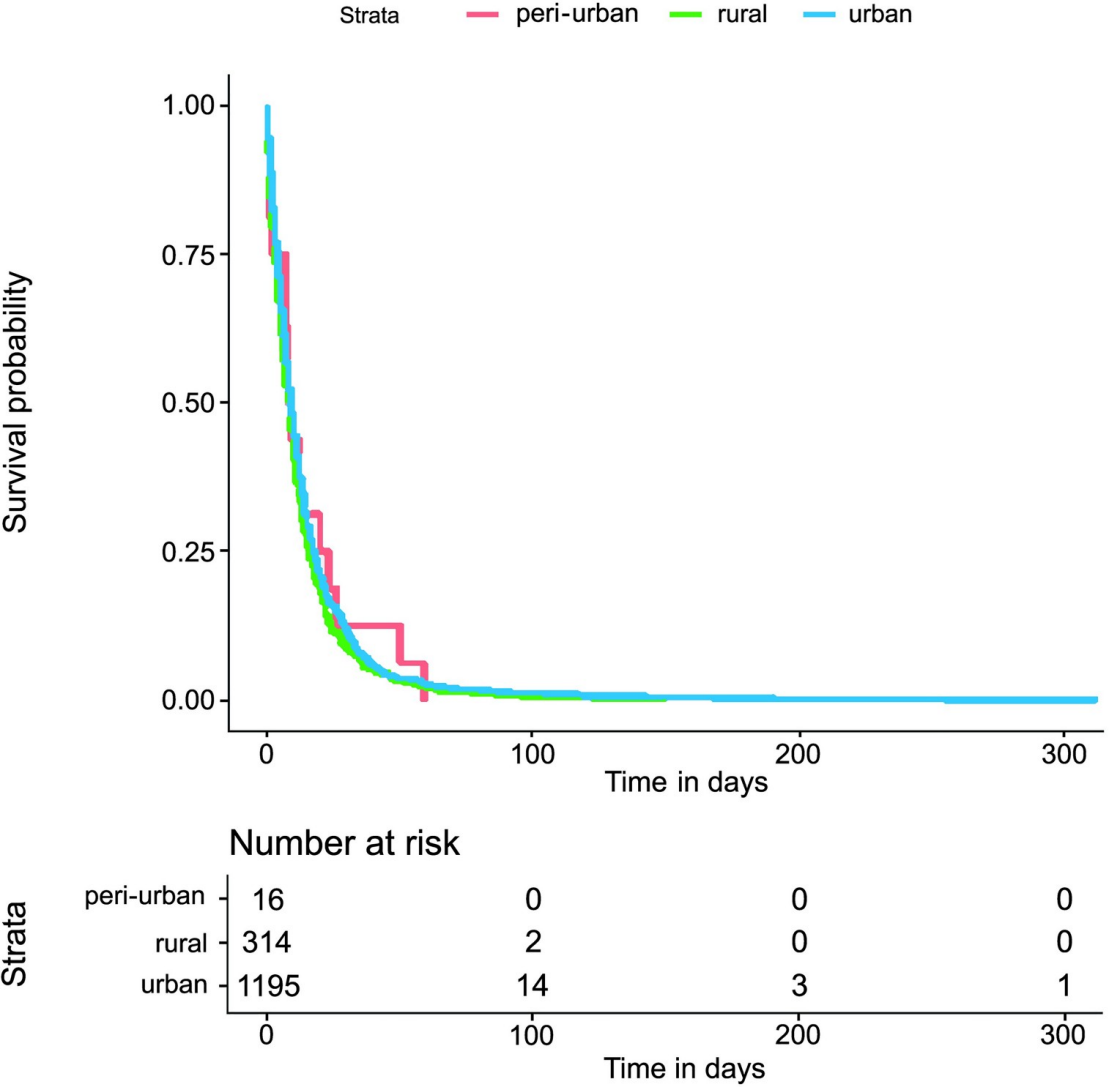
Kaplan−Meier survival estimate for the time period from the notification to the death (tNotD) according to the area of living.

The Kaplan−Meier survival estimate graph presented in Figs 2 and 3 revealed apparent differences between the time to event investigations and HIV status, being greater for HIV infected individuals particularly in the tNotD. Indeed, the median tNotD for HIV positives is 13 days (interquartile range (IQR) 6–26), while the median for the HIV negatives is 9 days, (IQR 4–17). In addition, in these explorations, the tStoN appears to be greater in peri-urban and rural areas compared to the urban areas, feature evident the Kaplan−Meier presented in Fig 4. The median tStoN for urban areas is 23 days (IQR, 10–59), while the median for the peri-urban is 46 days, IQR 21–92 and the median for the rural is 29 days (IQR 10–65). On the other hand, no difference was observed in the tNotD (Fig 5).

**Fig 6:**
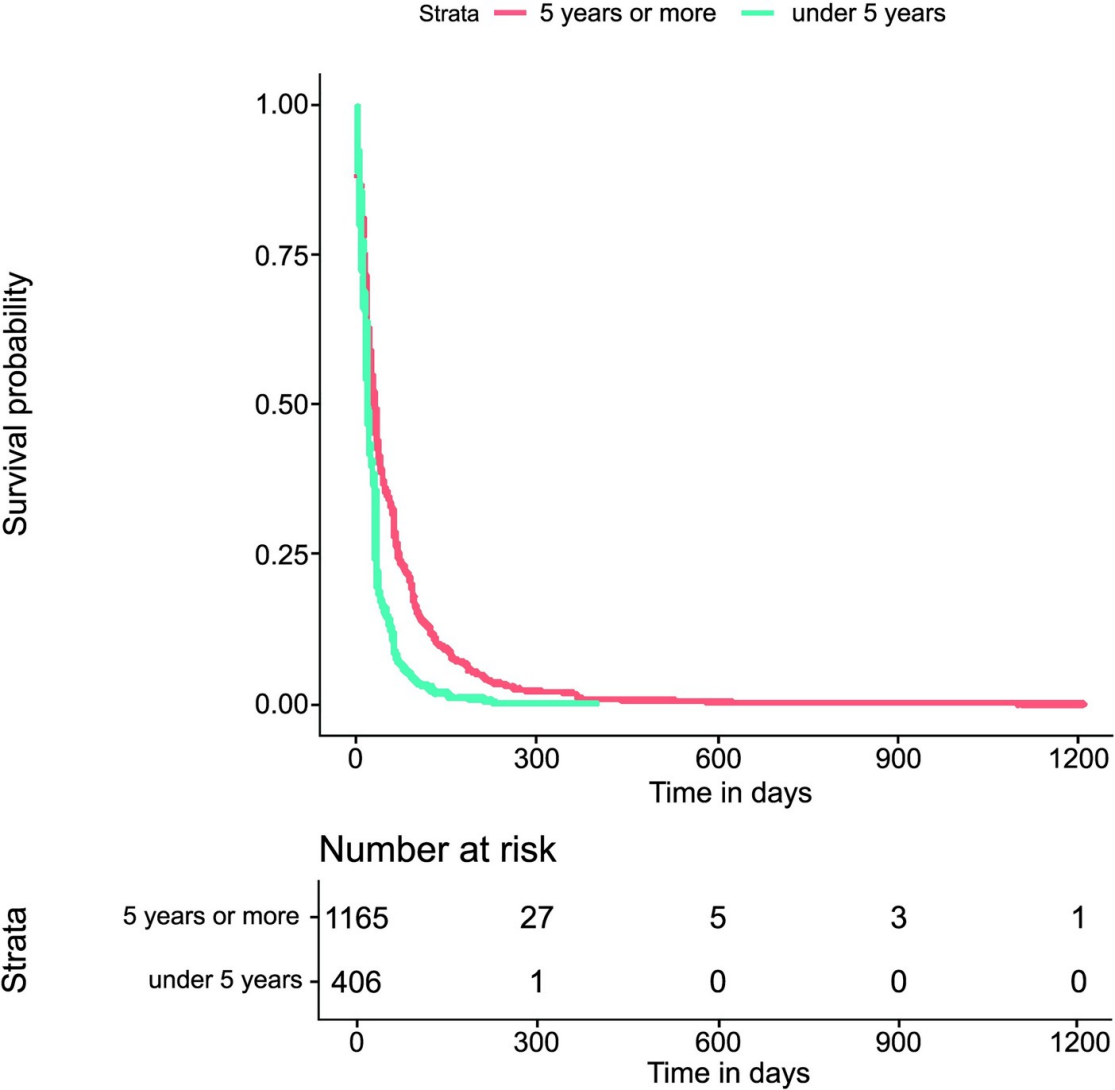
Kaplan−Meier survival estimate for the time period from the onset of the symptoms to the notification of the disease (tStoN) according to the age at the onset of the first symptoms.

**Fig 7:**
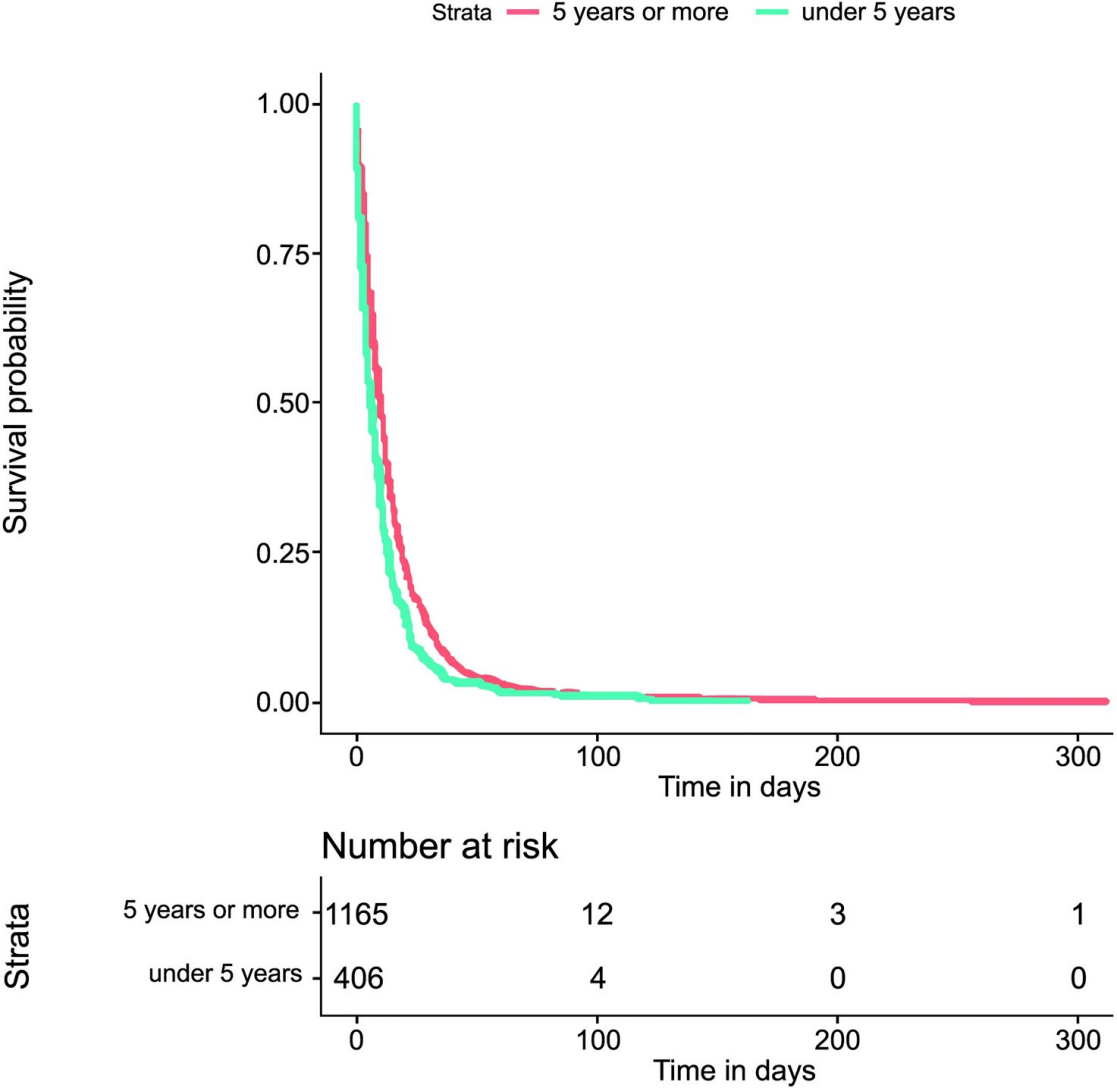
Kaplan−Meier survival estimate for the time period from the notification to the death (tNotD) according to the age at the onset of the first symptoms.

**Fig 8:**
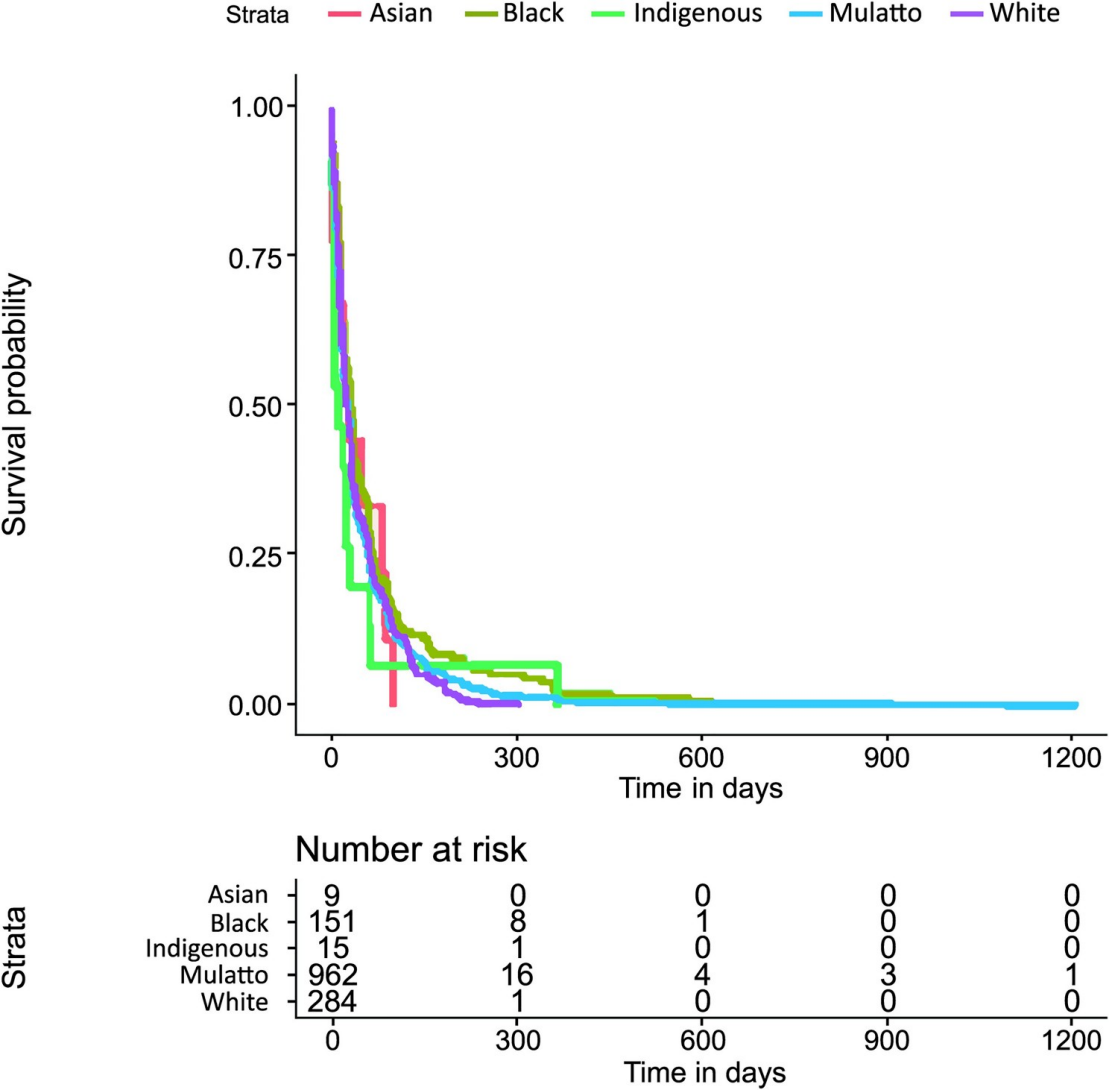
Kaplan−Meier survival estimate for the time period from the onset of the symptoms to the notification of the disease (tStoN) according to the ethnicity.

**Fig 9:**
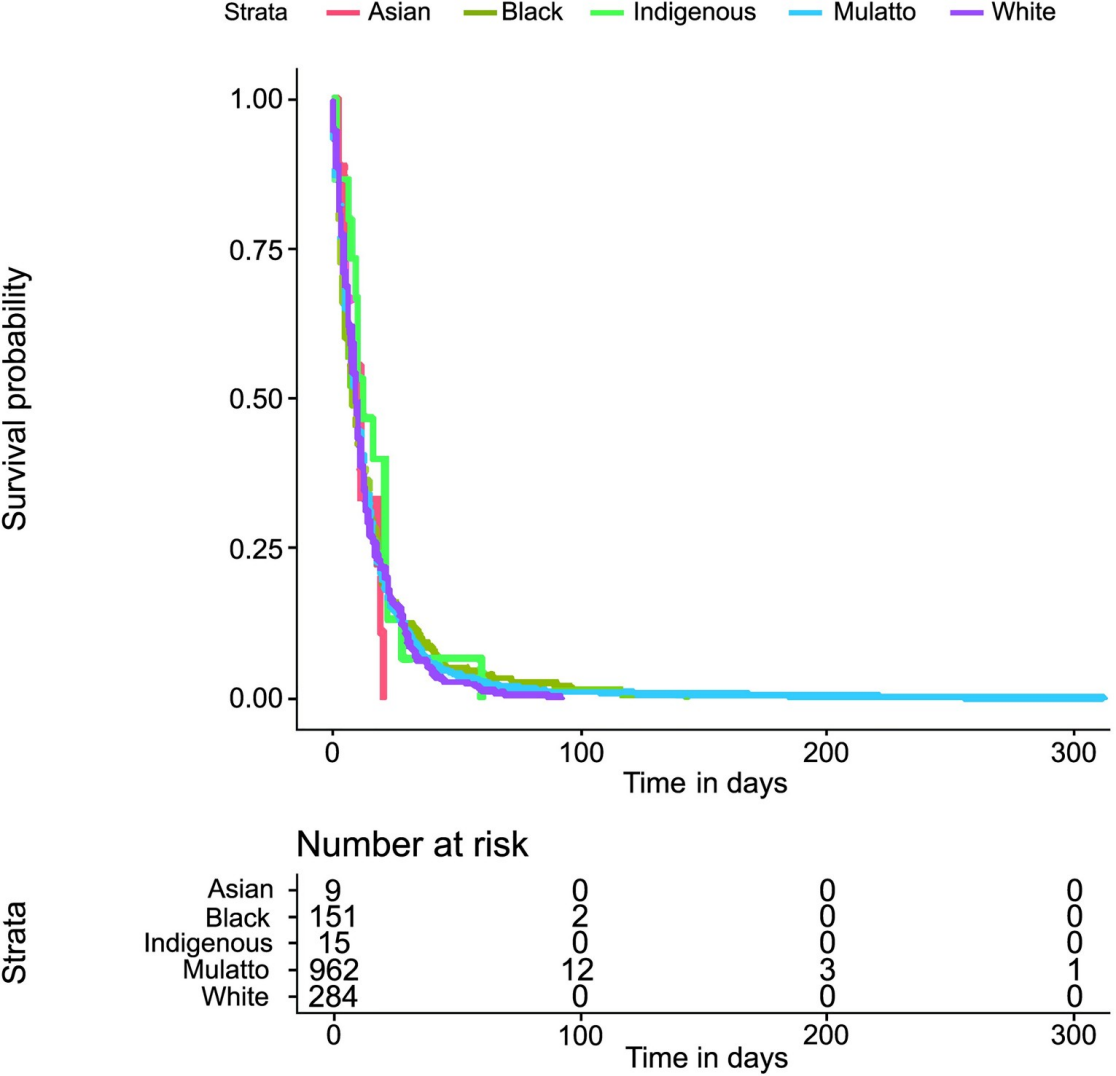
Kaplan−Meier survival estimate for the time period from the notification to the death (tNotD) according to the ethnicity.

The Kaplan−Meier survival estimate of the times to events investigation (both for tStoN and tNotD) according to the age was explored through a two-fold approach: first, splitting the age into three categories (under one year old, from one to five years old and over five years old); and second, splitting the age into two categories (under five years old and over five years old). In these explorations, there were not evident differences in the times to events between the categories under one year old and that from one to five years, and therefore only the investigation with two categories (under five years old and over five years old) was kept. Thus, this exploration revealed apparent differences between the time to event investigation (both for tStoN and tNotD) and the age at the onset of the symptoms (Figs 6 and 7), demonstrating that the time to event in both cases was greater in patients over 5 years old. In fact, the median tStoN for patients under 5 years old is 17 days (IQR 8–31), while the median for patients over 5 years is 28 days (IQR 11–68). Additionally, the median tNotD for patients under 5 years old is 6 days (IQR 2–13), while the median for patients over 5 years is 10 days (IQR 4–19). Concerning the ethnicity, it is observed in the Kaplan−Meier graphs that indigenous patients were notified earlier compared to other ethnic groups (Fig 8); however, no apparent differences were observed in the tNotD, (Fig 9). In the time to event distribution across the years; the difference across the years appeared to be subtle and do not seems to follow any pattern.

### Cox proportional hazards model

The unadjusted results for the different variables and the results of the final models are presented in Tables 2 and 3. Thus, we observed that at any time the ratio of notification (according to the tStoN) is 1.73 times greater in patients under 5 years old at the onset of the clinical symptoms compared to older patients (Table 2). In short, the notification is faster in the younger group. Likewise, the urban area also appears to be associated as a factor to reduce the time of notification. Similarly, as observed in the Table 3, once the disease is reported, death overcomes faster in the younger group; while the HIV negative group also has a significant greater death ratio when compared to the HIV positive group.

**Table 2:** Estimated hazard ratio (adjusted and unadjusted) for the covariates in the multivariable Cox proportional hazards regression model for the time between the date on the onset of the clinical signs and the date of notification, 2007–2014.

Predictor	Hazard Ratio Unadjusted (95% CI)	Hazard Ratio Adjusted (95% CI)
Age (under 5 years old compared to 5 or more)	1.67 (1.48–1.87)	1.73 (1.52–1.96)
Area (relative to rural)		-
Peri-urban	0.8 (0.48–1.31)	0.83 (0.47–1.44)
Urban	1.16 (1.03–1.31)	1.33 (1.16–1.52)
Ethnicity (relative to black)		
Asian	1.31 (0.67–2.56)	1.08 (0.51–2.31)
Indigenous	1.65 (0.97–2.8)	3.24 (1.74–6.05)
*Mulatto*	1.2 (1.01–1.42)	1.14 (0.96–1.36)
White	1.24 (1.02–1.51)	1.19 (0.97–1.46)

**Table 3:** Estimated hazard ratio (adjusted and unadjusted) for the covariates in the multivariable Cox proportional hazards regression model for the time between the date of the notification and the date of death, 2007–2014.

Predictor	Hazard Ratio Unadjusted (95% CI)	Hazard Ratio Adjusted (95% CI)
Age (under 5 years old relative to 5 or more)	1.48 (1.24–1.78)	1.25 (1.09–1.43)
HIV (negative status relative to positive status)	1.38 (1.23–1.54)	1.4 (1.16−1.68)

These models satisfied the Cox proportional hazards assumption of the independence between residuals and time, as it was observed in the Schönfeld residuals where the mean of residuals remains constant across time.

The results of the sensitivity analyses (without including the observations with zero value) presented in the S1 Supporting Information are very similar (and for some estimates almost identical) to those obtained in the models with the full datasets presented in the Tables 2 and 3; which indicates a very low, or perhaps even negligible, influence of those observations with zero value in these results.

## Discussion

There are some reports on host factors associated to death by VL and some signs and symptoms have been described as predictors of a poor outcome. However, very few studies have been performed with the objective of identifying factors related to social issues, race and mainly factors imputed to VL control programs that could have a role influencing a lethal outcome by VL.

This study was set up as a survival analysis as it aimed at the identification of factors that were able to influence the VL patient “survival” time to incident, for notification and for death. Initially, analyzing data of 1,589 deaths by VL we observed an annual mean of 198.6 deaths per year, without difference between the years. Unfortunately, the decision taken by the VL control program to promote earlier diagnosis, and certainly more accurate treatment, was not reflected in a mortality reduction. Some factors could contribute to maintenance of constant fatality over the years. Firstly, a long time elapsed between the onset of symptoms and the diagnosis and initiation of treatment could predict an unfavorable outcome. Nonetheless, we observed the time to incident investigated had a peak in the zero, indicating that in many cases, the notification date is also given as the date for the onset of the clinical signs in the Sinan-NET and likewise the date of death is given as date for notification in many cases. These zero inflated “artefacts” in the official records had no influence in the results of Cox proportional hazards regression multivariate models, as a constant mean of residuals across time in the plot of Schönfeld confirms that the proportional hazard assumption holds for the covariates. Moreover, the sensitivity analysis performed, without including the observations with zero value, obtained very similar results. Certainly, there is a bias regarding the interpretation of these results, since the onset of symptoms should be prior to the time of the VL diagnosis. In fact, the improvement in the quality of completing the complaint report is a fact that must be taken into account in order to provide data that are closer to the reality and could actually determine if the longest time between onset of symptoms and the diagnosis had an influence on the outcome of VL. A supplementary analysis of the death cases, excluding those with time since notification to death equal to “0”, failed to demonstrate relevant differences in the HR estimations for the predictors included in the final model. Then, we maintained the HR estimations obtained with the total sample.

On the other hand, the information system has intrinsic limitations. Clearly, data obtained from the surveillance notification system could be imprecise to determine both the time to notification and the time to death under analysis in the present research. This could be attributed to the memory bias and the lack of opportune notification of suspected cases, explaining the relevant number of notifications with time to death equal to 0, a very implausible scenario. Despite those limitations, the factors associated with the outcomes being explored in the present study seem to be robust because they remained basically unchanged after exclusion of the records with time to death data equal to 0, as stated above.

Several factors could be associated to death by VL, as some symptoms, age and toxicity due to specific treatment. Fever over 60 days has been considered an important factor related to death by VL [19] and frequently associated to delayed diagnosis [35]. They also identified that time between the onset of symptoms and the therapeutic intervention greater than 60 days, was associated with higher lethality [35].

Curiously, our results revealed that patients under 5 years of age were diagnosed earlier when compared to older patients and had a shorter survival. It could mean that in this younger population, although properly diagnosed, the lethality pattern might be related to the greater severity of the disease. Some host characteristics were evaluated and seem to have an impact in the disease evolution, as age, co-infections and ethnicity. The genetic background of the host influences the progression of the disease, from asymptomatic to severe VL outcomes. For example, a population genetics study provided evidence for the link between TGF beta pathway and progression of *L*. *infantum* infection [36]. Recently it was shown that clinical severity of VL is associated with changes in the profile of IgG Fc N-Glycopepetide and that this is restored after treatment [37]. However, the predictors considered in the present study are not approached in studies focusing on host genetics and VL progression.

The parasite is among the factors that might be contributing for VL progression. In Brazil, *L*. *infantum*, a viscerotropic parasite, is the main agent of VL. Visceral *Leishmania* species withstand higher temperatures than cutaneous species; the latter considered more sensitive to heat shock. Fever in visceral leishmaniasis exceeds 40°C, (it can vary among patients), variation in the heat shock susceptibility among *L*. *infantum* strains might contribute to the parasite survival and proliferation in visceral organs, but this variability was never investigated. Furthermore, *L*. *infantum* strains sensitive and resistant to nitric oxide (NO) were already observed infecting humans in Brazil [38]. Fever contributes to increasing oxidant production by phagocytic cells [39] and parasites more resistant to NO will have advantage in this condition. The association between resistant NO *L*. *infantum* strains and the severity of VL remains to be investigated.

Several other factors have been associated to death in VL patients, among which stands out edema, jaundice, weakness, *Leishmania*-HIV co-infection, other associated infections, bleeding, diarrhea, anemia, platelets < 50.000, leucopenia and age 50–60 years old [18]. The early recognition of these factors could contribute to the decrease of lethality, by the identification of more susceptible patient likely to have an unfavorable outcome. Some early symptoms can be associated with death. A previous study found that weakness is associated to death in VL, therefore, this factor should be investigated at the initial phase of patients with suspected VL, considering that it is an early marker of unfavorable outcome [40].

In a meta-analysis, looking for risk factors for adverse prognostic and death by American Visceral Leishmaniasis, did not observe prolonged fever was not observed as a risk to death by VL, but some other symptoms could be related to unfavorable outcomes, such as: jaundice and HIV coinfection [18].

*Leishmania infantum* in Brazil is composed by at least three populations, one widespread in the Country and the other two more concentrated in the Central-West [27]. All the genetic analyses indicate a tendency of geographical aggregation of *L*. *infantum* genotypes in Brazil. If the parasite has a role in the clinical outcome, the location of infection should be assessed as a predictor, but unfortunately, the available data did not allow this analysis.

It is also interesting to mention that recently a study conducted with patients from Piauí demonstrated that the parasite burden is higher in children **<**1year old, which drops sharply after the first year of life and remains lower until adolescence. Following adolescence, the parasite burden increases with age, peaking among elderly individuals. Men have a higher parasite burden than women. HIV-infected patients had a much higher parasite burden than non-infected patients. Interestingly, it seems that parasite burden is not a factor contributing to the time from the first symptoms to death [41]

Our findings identified that children under 5 years old are at higher risk of premature death being very relevant for planning interventions aimed at VL lethality reduction. Despite the physio pathological processes linked to host vulnerability or related to parasite virulence, the opportunity for effective interventions to avoid lethal outcomes in children should be prioritized. As we demonstrated, a very short period is available for any intervention, and readily access to efficient health facilities could be crucial. In 2009, the rK39 –immunochromatographic test (rK39—ICT) was introduced in Brazil for diagnosis of human VL with serum samples. At first, this test was available at the Central Laboratories and Reference Centers in all Brazilian states. However, as of 2015 this test was replaced by a new RDT, allowing testing of serum, plasma and whole blood samples, which permitted the testing of people at their hospital bed. Given that this new RDT is more practical and faster, the country has been working on a strategy, along with all the states and municipalities to decentralize and utilize this test at primary health care units [13]. Quality of care offered to the pediatric population should be considered because of the shorter time between diagnosis (notification) and death in younger children. This is a relevant issue considering that a relatively small number of VL pediatric patients are dispersed across a large territory being difficult to maintain well trained teams for the effective and opportune detection and treatment of this vulnerable population. Although it could be a paradox, centralized attention of VL patients should be considered as it is currently thought for some rare diseases with referral centers capable of know-how accumulation on improving prognosis. Our results suggest that cases from urban areas had shorter time between the onset of symptoms and case notification as was expected considering that in general urban areas could have better access to healthcare facilities. On the other hand, peri urban cases seem to be similar to rural ones (Adjusted HR 0.83; CI95% 0.47 to 1.44) showing that access problems could be also relevant for this population. It underscores the need of in-depth investigation on patients’ itineraries looking for healthcare in order to solve the gaps precluding opportune diagnosis and treatment.

Otherwise, the HIV coinfected VL patients had lower hazard to death compared to the HIV negative VL cases. Higher fatality rates have been widely recognized among HIV-VL coinfected patients [42] but the survival time from diagnosis to death, as far as we know, has been never explored before as we did in the present report. Our finding could be explained because coinfected adults have been cared at referral treatment centers for HIV/AIDS by infectologists that are reasonably aware of HIV-VL coinfection, offering opportune treatment and toxicity monitoring and management, which is unusual in other clinical settings. Moreover, the use of secondary prophylaxis is expected to avoid some relapses prolonging survival but not to avoid a later lethal outcome.

As the whole therapeutic options against VL is composed by toxic medications, toxicity management should be taken into consideration when discussing lethality. Until the development of better drugs, toxicity monitoring and prevention should be prioritized to decrease current fatality rates.

Adequate management of toxicity would be possible in the in-patient clinical settings despite higher direct costs. The cost of VL has been recently estimated in Brazil and the most important component is related to the loss of productivity due to premature death [43], therefore, avoiding toxicity through proper care could be economically advantageous in the decreasing of lethality. Finally, we need to implement large-scaled pragmatic clinical trials in VL patients having death as the primary outcome. Current data obtained from the surveillance systems will not respond satisfactorily on the prognostic factors that should be addressed for the effective improvement of the interventions needed to achieve lower fatality rates. Poverty and inequality indicators along with healthcare access measures should be incorporated in the prognosis analysis of such trials in order to never forget the economic background of this lethal disease.

## Supporting information

S1 Supporting information Analysis without the zeros to evaluate the consistency of the results presented in the main text in relation to the influence of the observation with the zero values in the two time to event periods investigated (the time between the date on the onset of the symptoms and the date of notification, tStoN, and time between the date of the notification and the date of death, tNotD). (DOCX)

S2 Supporting information STROBE Statement—Checklist of items for reports of cohort studies. (DOCX)
