# Supplementary material for: Premature deaths by visceral leishmaniasis in Brazil investigated through a cohort study: A challenging opportunity?
Source: PLoS Negl Trop Dis. 2019 Dec 19;13(12):e0007841. doi: 10.1371/journal.pntd.0007841 (PMC6922316; doi:10.1371/journal.pntd.0007841)
Supplement: S1 Supporting information — (DOCX) [file pntd.0007841.s001.docx]

**Supporting information 1. Analysis without the zeros.**

As a way of a sensitivity analysis to evaluate the consistency of the results presented in the main text in relation to the influence of the observation with the zero values in the two time to event periods investigated (the time between the date on the onset of the symptoms and the date of reporting, tStoN, and time between the date of the reporting and the date of death, tNotD). That is, for tStoN those in which the date of the notification was the same as the data of the onset of the symptoms, and for tNotD those in which the date of the death was the same as data of the notification.

**Data Source**

As described in the main text, all registered deaths by VL (defined as: deaths directly associated to the infection by *Leishmania infantum* or to complications of this infection) were sourced from the Brazilian National Notification System (Sinan-NET, acronym in Portuguese) from 2007 through 2014. The system holds individual information on VL cases, including deaths, such as: date of birth, onset of the first clinical signs, date of the disease notification and date of death. Additionally, we also gathered information on gender, HIV status, area of residence and ethnic group, which were also available in the Sinan-NET.

**Material and methods**

The two different time periods (tStoN and tNotD) were analyzed as survival models to investigate different factors available (the predictors) for the study (i.e. HIV status, gender, area of residence, age at the onset of the VL first symptoms and year of the notification) that might be influencing the time to incident (notification and death). Thus, two separate multivariable Cox proportional hazard models, one for tStoN and one for tNotD, were computed to investigate the effect of the different predictive variables on the time to incident (reporting or death). There were not censoring events considered in these models, since both incidents investigated in the models (reporting and death) occurred for all the observations. Given the small number of investigated predictors, all of them were attempted into a multivariable Cox proportional hazards model using a Wald stepwise process, and covariates were kept if they met a P-value <0.05 as significant criteria. Once final multivariable models were developed, validity of assumption that the effects of predictors (covariates) are constant over time for Cox proportional hazards models were tested (Cox, 1972). To do so the scaled Schönfeld (Grambsh, and Therneau, 1994) residuals were plotted against time together with a smooth curve that helped with the interpretation of the result.

All the above-mentioned analyses were performed with the R version 2.5.1 from the R Foundation for Statistical Computing. <http://www.r_project.org>, library (survival)

**Results**

The results are very similar (and for some estimates almost identical) to those obtained in the models with the full datasets presented in the Tables 2 and 3 of the main text; which indicates a low, or perhaps even negligible, influence of those observations with zero value.

**Table S1. Estimated hazard ratio (adjusted and unadjusted) for the covariates in the multivariable Cox proportional hazards regression model for the time between the date on the onset of the clinical signs and the date of reporting (tStoN), 2007–2014. n=1259 (original dataset (n= 1355) without the observations with a zero value for the for the tStoN)**

| Predictor |  | Hazard Ratio  Adjusted (95% CI) |
| --- | --- | --- |
| Age (under 5 years old relative to 5 or more) |  | 1.84 (1.61-2.1) |
| Area (relative to rural) |  |  |
| Peri-urban |  | 0.89 (0.51-1.56) |
| Urban |  | 1.39 (1.21-1.6) |
| Ethnicity (relative to black) |  |  |
| Asian |  | 0.97 (0.43-2.2) |
| Indigenous |  | 3.22 (1.62-6.38) |
| Mulatto |  | 1.12 (0.93-1.34) |
| White |  | 1.18 (0.95-1.45) |
|  |  |  |

**Table S2. Estimated hazard ratio (adjusted and unadjusted) for the covariates in the multivariable Cox proportional hazards regression model for the time between the date of the reporting and the date of death (tNotD), 2007–2014. n=1051 (original dataset n= 1112) without the observations with a zero value for the for tNotD)**

| Predictor |  | Hazard Ratio  Adjusted (95% CI) |
| --- | --- | --- |
| Age (under 5 years old relative to 5 or more) |  | 1.17 (1.02-1.36) |
| HIV (negative status relative to positive status) |  | 1.4 (1.16-1.69) |
|  |  |  |
