# Supplementary material for: Premature deaths by visceral leishmaniasis in Brazil investigated through a cohort study: A challenging opportunity?
Source: PLoS Negl Trop Dis. 2019 Dec 19;13(12):e0007841. doi: 10.1371/journal.pntd.0007841 (PMC6922316; doi:10.1371/journal.pntd.0007841)
Supplement: S2 Supporting information — (DOCX) [file pntd.0007841.s002.docx]

STROBE Statement—Checklist of items that should be included in reports of ***cohort studies***

|  | Item No | Recommendation |
| --- | --- | --- |
| **Title and abstract** | 1 | (*a*) Indicate the study’s design with a commonly used term in the title or the abstract |
|  |  | **Premature deaths from visceral leishmaniasis in Brazil investigated through a cohort study: a challenging opportunity?** |
|  |  | (*b*) Provide in the abstract an informative and balanced summary of what was done and what was found  **Through a retrospective cohort study, we investigated the factors that could influence the time elapsed between the onset of the first symptoms of VL to death, and univariate analyses were performed. (found in abstract and title)** |
| Introduction | | |
| Background/rationale | 2 | Explain the scientific background and rationale for the investigation being reported  **Done (Introduction – objectives)** |
| Objectives | 3 | State specific objectives, including any prespecified hypotheses  **Done (Introduction – objectives)** |
| Methods | | |
| Study design | 4 | Present key elements of study design early in the paper  **Study design indicated at the beginning of M & M (Methods – study design).** |
| Setting | 5 | Describe the setting, locations, and relevant dates, including periods of recruitment, exposure, follow-up, and data collection  **Indicated in data source** **(Methods – setting).** |
| Participants | 6 | (*a*) Give the eligibility criteria, and the sources and methods of selection of participants. Describe methods of follow-up  **Indicated in data source (Methods – participants).** |
|  |  | (*b*) For matched studies, give matching criteria and number of exposed and unexposed  **No applicable** |
| Variables | 7 | Clearly define all outcomes, exposures, predictors, potential confounders, and effect modifiers. Give diagnostic criteria, if applicable  **Explained with the section about variables** **(Methods – variables).** |
| Data sources/ measurement | 8* | For each variable of interest, give sources of data and details of methods of assessment (measurement). Describe comparability of assessment methods if there is more than one group  **Explained with the section about variables and data source (Methods – data source).** |
| Bias | 9 | Describe any efforts to address potential sources of bias  **168 deaths were excluded from this analysis due to data inconsistency and unavailability.**  **It was evaluated the consistency of the results in relation to the influence of the observation with the zero values in the two time to event periods investigated (tStoN and tNotD). The results of this analyses (without including the observations with zero value) presented in the supporting information 1 of this article are very similar (and for some estimates almost identical) to those obtained in the models with the full datasets presented in the Tables 2 and 3; which indicates a very low, or perhaps even negligible, influence of those observations with zero value in these results. (Methods – Bias).** |
| Study size | 10 | Explain how the study size was arrived at  **This is a retrospective study utilizing existing data, so no sample size was calculated. Moreover, the number of observations in the dataset utilized was very large, 1,589 deaths by visceral leishmaniasis.** **(Methods – study size).** |
| Quantitative variables | 11 | Explain how quantitative variables were handled in the analyses. If applicable, describe which groupings were chosen and why  **The categorization of age is explained in the results section.**  **(Methods – data source).** |
| Statistical methods | 12 | (*a*) Describe all statistical methods, including those used to control for confounding  **Described in section Exploratory and Statistical Analyses** **(Methods).** |
|  |  | (*b*) Describe any methods used to examine subgroups and interactions  **Described in section Exploratory and Statistical Analyses** **(Methods).** |
|  |  | (*c*) Explain how missing data were addressed  **NA** |
|  |  | (*d*) If applicable, explain how loss to follow-up was addressed  **NA** |
|  |  | (*e*) Describe any sensitivity analyses  **Described in section Exploratory and Statistical Analyses (Methods).** |
| Results | | |
| Participants | 13* | (a) Report numbers of individuals at each stage of study—eg numbers potentially eligible, examined for eligibility, confirmed eligible, included in the study, completing follow-up, and analysed  **Done in data source and exploratory analysis results section (Results).** |
|  |  | (b) Give reasons for non-participation at each stage  **NA** |
|  |  | (c) Consider use of a flow diagram  **Not necessary** |
| Descriptive data | 14* | (a) Give characteristics of study participants (eg demographic, clinical, social) and information on exposures and potential confounders  **Done on the section of results of exploratory analyses** **(Descriptive data).** |
|  |  | (b) Indicate number of participants with missing data for each variable of interest  **Done on the section of results of exploratory analyses Kaplan−Meier survival curves including the individuals at risk table (Descriptive data).** |
|  |  | (c) Summarise follow-up time (eg, average and total amount)  **Done on the section of results of exploratory analyses Kaplan−Meier survival curves including the individuals at risk table** **(Descriptive data).** |
| Outcome data | 15* | Report numbers of outcome events or summary measures over time  **Done on the section of results of exploratory analyses Kaplan−Meier survival curves including the individuals at risk table (Outcome data).** |
| Main results | 16 | (*a*) Give unadjusted estimates and, if applicable, confounder-adjusted estimates and their precision (eg, 95% confidence interval). Make clear which confounders were adjusted for and why they were included  **Done in the results of the multivariable Cox proportional hazard models (main results).** |
|  |  | (*b*) Report category boundaries when continuous variables were categorized  **Done in the results of the multivariable Cox proportional hazard models (main results).** |
|  |  | (*c*) If relevant, consider translating estimates of relative risk into absolute risk for a meaningful time period  **NA** |
| Other analyses | 17 | Report other analyses done—eg analyses of subgroups and interactions, and sensitivity analyses  **Reported in the supplementary material 1** |
| Discussion | | |
| Key results | 18 | Summarise key results with reference to study objectives  **The study revealed apparent differences between the time to event investigations and HIV status, being greater for HIV infected individuals particularly in the tNotD.**  **The time (tStoN) is 1.73 times greater in patients under 5 years old at the onset of the clinical symptoms compared to older patients.**  **(Discussion).** |
| Limitations | 19 | Discuss limitations of the study, taking into account sources of potential bias or imprecision. Discuss both direction and magnitude of any potential bias.  **For this study we used secondary data from the Brazilian Leishmaniasis surveillance system and there was no verification and comparison of these deaths with that of the official mortality system. On the other hand, a study already carried out shows that this official mortality system presents a sub-notification of deaths by VL in comparison with the deaths recorded in the system of recording morbidity**  **(Discussion).** |
| Interpretation | 20 | Give a cautious overall interpretation of results considering objectives, limitations, multiplicity of analyses, results from similar studies, and other relevant evidence  **We observed that the time (tStoN) is 1.73 times greater in patients 5 years old at the onset of clinical symptoms compared to older patients. On the other hand, survival (tNotD.) in under 5 years of age is also lower than in those older than 5 years. The urban area also seems to be associated as a factor to reduce reporting time. As observed in our study, since the disease is reported, death outweighs more rapidly in the younger group; While the HIV-negative group also has a significant higher death ratio when compared to the HIV-positive group. This last result shows a very relevant data, because many other studies show that HIV-positive are risk factors for death.**  **(Discussion).** |
| Generalisability | 21 | Discuss the generalisability (external validity) of the study results.  **Brazil represents 96% of the cases of LV of the Americas and these results represent an internal validity for this region, and should not be extrapolated to other continents, due to the differences between the epidemiology of the disease, the characteristics of the population, the Organization of health services, among others.**  **(N/A).** |
| Other information | | |
| Funding | 22 | Give the source of funding and the role of the funders for the present study and, if applicable, for the original study on which the present article is based  **This study had no funding** |

*Give information separately for exposed and unexposed groups.

**Note:** An Explanation and Elaboration article discusses each checklist item and gives methodological background and published examples of transparent reporting. The STROBE checklist is best used in conjunction with this article (freely available on the Web sites of PLoS Medicine at http://www.plosmedicine.org/, Annals of Internal Medicine at http://www.annals.org/, and Epidemiology at http://www.epidem.com/). Information on the STROBE Initiative is available at http://www.strobe-statement.org.
